# Antifungal Susceptibility and Mutations in the Squalene Epoxidase Gene in Dermatophytes of the Trichophyton mentagrophytes Species Complex

**DOI:** 10.1128/AAC.00056-21

**Published:** 2021-07-16

**Authors:** Xue Kong, Chao Tang, Ashutosh Singh, Sarah A. Ahmed, Abdullah M. S. Al-Hatmi, Anuradha Chowdhary, Pietro Nenoff, Yvonne Gräser, Steven Hainsworth, Ping Zhan, Jacques F. Meis, Paul E. Verweij, Weida Liu, G. Sybren de Hoog

**Affiliations:** a Department of Medical Mycology, Institute of Dermatology, Chinese Academy of Medical Science & Peking Union Medical College, Nanjing, China; b Center of Expertise in Mycology Radboud University Medical Center/Canisius Wilhelmina Hospital, Nijmegen, The Netherlands; c Medical Mycology Unit, Department of Microbiology, Vallabhbhai Patel Chest Institute, University of Delhi, Delhi, India; d Natural & Medical Sciences Research Center, University of Nizwa, Nizwa, Oman; e Laboratory for Medical Microbiology, Mölbis, Germany; f Universitätsmedizin Berlin-Charité, Institut für Mikrobiologie und Hygiene, Nationales Konsiliarlabor für Dermatophyten, Berlin, Germany; g School of Science, RMIT University (Bundoora Campus), Bundoora, Australia; h Dermatology Hospital of Jiangxi Provinces, Jiangxi Dermatology Institute, Nanchang, China; i Department of Medical Microbiology and Infectious Diseases, Canisius-Wilhelmina Hospital, Nijmegen, The Netherlands; j Center for Global Health, School of Public Health, Nanjing Medical University, Nanjing, China; k Jiangsu Key Laboratory of Molecular Biology for Skin Diseases and STIs, Nanjing, Jiangsu, China

**Keywords:** *Trichophyton mentagrophytes-T. interdigitale* complex, antifungal susceptibility testing, squalene epoxidase gene

## Abstract

During the past decade, a prolonged and serious outbreak of dermatophytosis due to a terbinafine-resistant novel species in the Trichophyton mentagrophytes*-*T. interdigitale complex has been ongoing in India, and it has spread to several European countries. The objective of this study was to investigate the molecular background of the squalene epoxidase (*SQLE*) gene in order to understand the risk of emergence and spread of multiresistance in dermatophytes. Antifungal susceptibility to fluconazole, griseofulvin, itraconazole, ketoconazole, miconazole, naftifine, sertaconazole, and terbinafine was tested in 135 isolates from India, China, Australia, Germany, and The Netherlands. Based on the latest taxonomic insights, strains were identified as three species: T. mentagrophytes *sensu stricto* (*n* = 35), T. indotineae (*n* = 64, representing the Indian clone), and T. interdigitale *sensu stricto* (*n* = 36). High MICs of terbinafine (>16 mg/liter) were found in 34 (53%) *T. indotineae* isolates. These isolates showed an amino acid substitution in the 397th position of the *SQLE* gene. Elevated MICs of terbinafine (0.5 mg/liter) were noted in 2 (3%) *T. indotineae* isolates; these isolates lead to Phe^415^Val and Leu^393^Ser of the *SQLE* gene. The stability of the effect of the mutations was proven by serial transfer on drug-free medium. Lys^276^Asn and Leu^419^Phe substitutions were found in susceptible T. mentagrophytes strains. The Phe^377^Leu/Ala^448^Thr double mutant showed higher MIC values for triazoles. High MICs of terbinafine are as yet limited to *T. indotineae* and are unlikely to be distributed throughout the T. mentagrophytes species complex by genetic exchange.

## INTRODUCTION

Dermatophyte infections are among the most frequent fungal disorders of humans worldwide. Classically, dermatophytoses are easily cured with a wide range of topical and oral antifungal drugs. However, the recent emergence of terbinafine (TBF)-resistant strains of the Trichophyton mentagrophytes species complex in India has raised concern (1). The strains were initially denominated “type VIII” based on ribosomal internal transcribed spacer (ITS) sequencing data. Confusion arose as to whether the strains should be identified as Trichophyton interdigitale (2–4) or T. mentagrophytes (3, 4). Whole-genome analysis of Indian T. mentagrophytes*-*T. interdigitale species complex isolates revealed that all these isolates were distinct from the T. mentagrophytes-T. interdigitale species of the genus (5). Consequently, Kano et al. (6) described a novel species, Trichophyton indotineae, which matches the genotype of the Indian T. mentagrophytes-T. interdigitale species complex. In addition, recent multilocus, phenotypic, and clinical data have shown that the maintenance of T. indotineae as a separate genotype is justified (7). *Trichophyton indotineae* has already spread to other countries such as Belgium (8), Germany (9), Japan (6, 10), Denmark (11), Iran (12), Finland (13), Switzerland (14), and Bahrain (15). The most likely scenario is that these resistant strains were imported from the Indian subcontinent and transmitted between humans. The origin and mechanisms of increasing terbinafine-resistant dermatophytes are not well understood. It has been suggested that wide abuse of over-the-counter topical steroid creams with terbinafine has triggered resistance, but a zoonotic origin might also be considered, which is not yet proven. The aims of our experiments are to (i) detect the frequency of terbinafine-resistant *Trichophyton* strains outside India, (ii) conduct antifungal susceptibility testing (AFST) based on the new taxonomy, (iii) establish whether resistance to terbinafine can be attributed to mutations in the squalene epoxidase (*SQLE*) gene, and (iv) evaluate the genetic stability of terbinafine resistance.

## RESULTS

### Identity and AFST.

**FIG 1:**
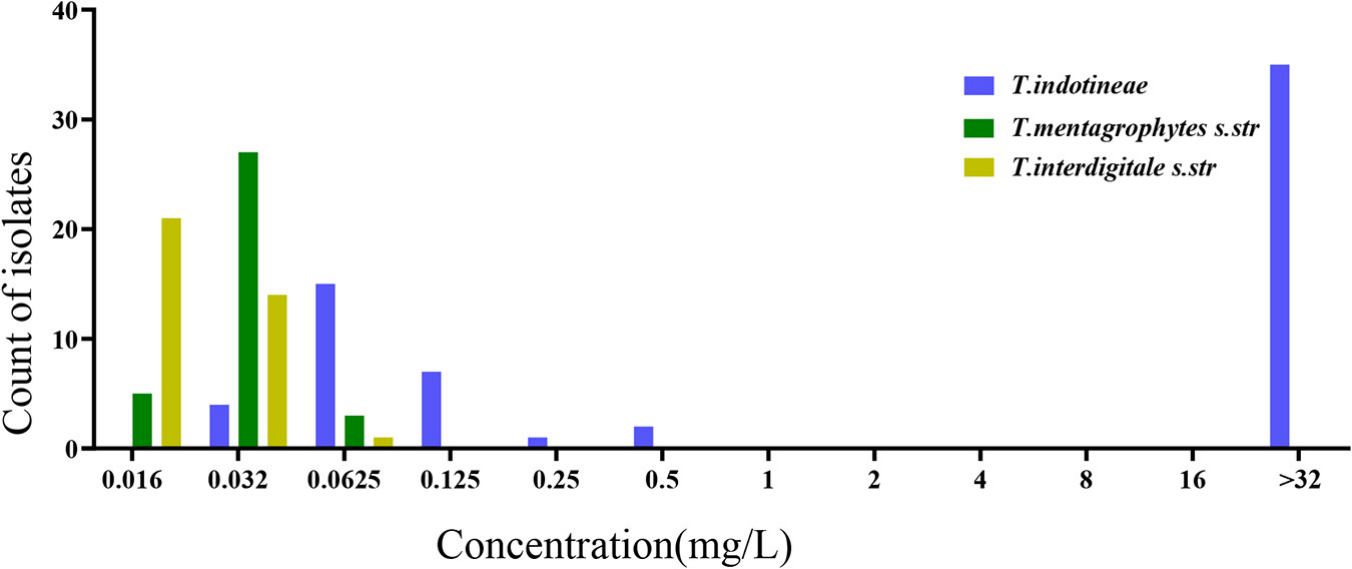
Distribution of terbinafine MIC values in different species. MICs of terbinafine in T. mentagrophytes and T. interdigitale do not exceed 0.125 mg/liter. Terbinafine-resistant strains are limited to *T. indotineae* *s.str.* (*sensu stricto*).

Using internal transcribed spacer (ITS) and high-mobility-group (HMG) sequencing, isolates were identified as *T. indotineae* (*n* = 64), T. interdigitale *sensu stricto* (*n* = 36), and T. mentagrophytes *sensu stricto* (*n* = 35). All strains from India were “T. mentagrophytes ITS type VIII,” now known as *T. indotineae*. *In vitro* antifungal susceptibility profiles of 135 *Trichophyton* isolates are summarized in Table 1. The geometric mean (GM) MICs for *T. indotineae* were observed to be 1.92 mg/liter for naftifine (NAF), 0.15 mg/liter for itraconazole (ITZ), 0.70 mg/liter for miconazole (MCZ), 0.37 mg/liter for ketoconazole (KTZ), 1.46 mg/liter for TBF, 1.16 mg/liter for sertaconazole (SCZ), 9.72 mg/liter for fluconazole (FCZ), and 3.96 mg/liter for griseofulvin (GRI); those for T. interdigitale were 0.06 mg/liter for NAF, 0.13 mg/liter for ITZ, 0.40 mg/liter for MCZ, 0.32 mg/liter for KTZ, 0.02 mg/liter for TBF, 1.47 mg/liter for SCZ, 1.92 mg/liter for FCZ, and 2.12 mg/liter for GRI; and those for T. mentagrophytes were 0.04 mg/liter for NAF, 0.17 mg/liter for ITZ, 0.44 mg/liter for MCZ, 0.23 mg/liter for KTZ, 0.03 mg/liter for TBF, 1.06 mg/liter for SCZ, 1.78 mg/liter for FCZ, and 5.94 mg/liter for GRI. All isolates identified as T. interdigitale and T. mentagrophytes were susceptible to TBF (MIC range, 0.016 to 0.0625 mg/liter; MIC_90_, 0.06 mg/liter). High MICs (>16 mg/liter) of TBF were noted in 34 (53%) *T. indotineae* isolates (Fig. 1). Elevated MICs of TBF (0.5 mg/liter) were noted in two isolates (3%), and low MICs of TBF (0.125 to 0.25 mg/liter) were noted in two strains (3%) of *T. indotineae*. All isolates with high TBF MICs also showed high MICs of NAF. *T. indotineae* had the highest GM (9.7 mg/liter) for FCZ, compared to T. interdigitale (GM of 1.9 mg/liter) and T. mentagrophytes (GM of 1.7 mg/liter). We performed a Kruskal-Wallis test comparing MIC values for FCZ among these three species, which yielded significant differences for *T. indotineae* compared with the other species (*H* = 46.347; *P* < 0.001). A total of 50 *Trichophyton* isolates were tested for the ability to grow on RPMI 1640 agar containing 0.2 mg/liter TBF and 4 mg/liter ITZ. All isolates with an MIC of ≥0.25 mg/liter of TBF (*n* = 37) grew well on RPMI 1640 agar containing 0.2 mg/liter TBF, while isolates with MICs of ≤0.125 mg/liter did not grow on this medium (see Table S2 in the supplemental material). None of the strains grew on RPMI 1640 agar containing ITZ.

**TABLE 1:** MIC distributions of *Trichophyton* species (*n* = 135) against eight drugs^*a*^

Species and parameter	Value (mg/liter)							
	NAF	ITZ	MCZ	KTZ	TBF	SCZ	FCZ	GRI
*T. indotineae* (*n* = 64)								
GM	1.92	0.15	0.70	0.37	1.46	1.16	9.72	3.96
MIC_50_	16.00	0.13	1.00	0.50	>16	1.00	16.00	4.00
MIC_90_	>16	0.25	2.00	1.00	>16	2.00	>32	8.00
Range	0.016 to >16	0.016 to 2	0.125 to 4	0.063 to 4	0.016 to >16	0.25 to 2	0.5 to >32	2 to >32

T. interdigitale (*n* = 36)								
GM	0.06	0.13	0.40	0.32	0.02	1.47	1.92	2.12
MIC_50_	0.06	0.13	0.25	0.25	0.02	2.00	2.00	2.00
MIC_90_	0.06	0.25	1.00	1.00	0.03	2.00	8.00	4.00
Range	0.031 to 0.125	0.031 to 0.5	0.0625 to 2	0.125 to 2	0.016 to 0.0625	0.25 to 4	0.25 to 16	0.25 to 8

T. mentagrophytes (*n* = 35)								
GM	0.04	0.17	0.44	0.23	0.03	1.06	1.78	5.94
MIC_50_	0.06	0.25	0.50	0.25	0.03	2.00	2.00	4.00
MIC_90_	0.06	1.00	2.00	1.00	0.03	4.00	16.00	>32
Range	0.016 to 0.0625	0.016 to 1	0.031 to 2	0.016 to 2	0.016 to 0.0625	0.0625 to 4	0.25 to 16	1 to >32

a NAF, naftifine; ITZ, itraconazole; MCZ, miconazole; KTZ, ketoconazole; TBF, terbinafine; SCZ, sertaconazole; FCZ, fluconazole; GRI, griseofulvin; GM, geometric mean.

### *SQLE* gene analysis.

**FIG 2:**
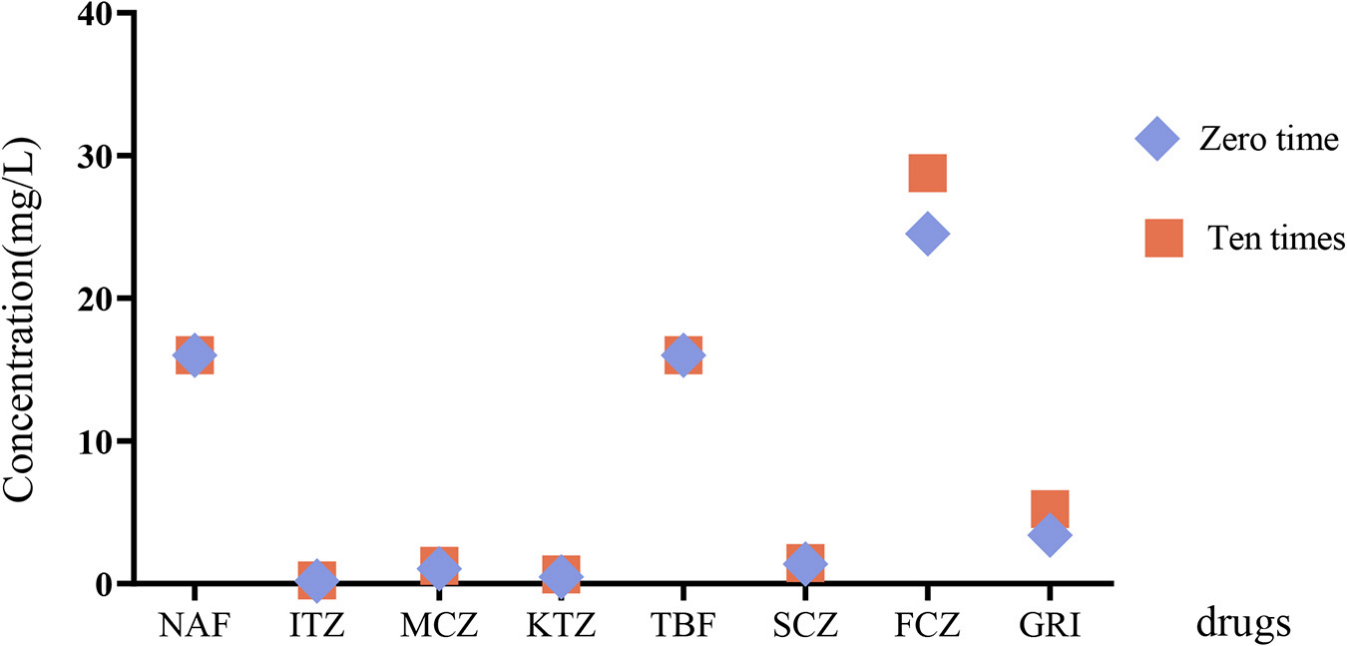
Comparison of AFST after 10 sequential passages on PDA drug-free medium. Geometric means of different drugs show no difference between 0 and 10 exposures.

Missense mutations leading to substituted amino acids in the SQLE protein were documented (Table 2). Thirty-four (53.13%) resistant *T. indotineae* strains had one of the previously recognized amino acid substitutions (Phe^397^Leu, mutation 1189T>C, 1191C>A, or 1191C>G) leading to a TBF-resistant phenotype and MICs of ≥16 mg/liter. Nine (26.5%) of these strains also carried an Ala^448^Thr substitution. Isolates with Phe^415^Val (*n* = 1) or Leu^393^Ser (*n* = 1) showed TBF MICs of 0.5 mg/liter. One isolate (MIC of 0.125 mg/liter) showed the mutation His^440^Tyr, but surprisingly, 15 other susceptible strains (MICs of 0.062 to 0.125 mg/liter) of *T. indotineae* had an additional specific amino acid substitution, Ala^448^Thr. Given the susceptible strains of *T. indotineae* with this mutation, we investigated whether other susceptible strains of T. mentagrophytes have the same amino acid substitution. We detected 10 strains from China with two undescribed missense mutations with substituted amino acids (Lys^276^Asn and Leu^419^Phe) in T. mentagrophytes; these strains had a susceptible phenotype (TBF MIC of 0.031 mg/liter). Comparison of AFST after 10 sequential passages on potato dextrose agar (PDA) drug-free medium revealed no difference in MIC ranges and GM values between 0 and 10 exposures (Table 3 and Fig. 2).

**TABLE 2:** Missense mutations in the coding region of the *SQLE* gene with amino acid substitutions in the SQLE enzyme

Missense mutation(s) of *SQLE*	Amino acid substitution(s)	GenBank accession no.	Origin	Genotype	MIC (mg/liter)	No. of strains	Frequency (%)
1189T>C	Phe^397^Leu	MW187980	India	*T. indotineae*	>16	23	35.90
1191C>A	Phe^397^Leu	MW188000	India	*T. indotineae*	>16	2	3.13
1189T>C, 1342G>A	Phe^397^Leu, Ala^448^Thr	MW187987	India	*T. indotineae*	>16	5	7.81
1191C>A, 1342G>A	Phe^397^Leu, Ala^448^Thr	MW187998	India	*T. indotineae*	>16	2	3.13
1191C>G, 1342G>A	Phe^397^Leu, Ala^448^Thr	MW188003	India	*T. indotineae*	>16	2	3.13
1243T>G	Phe^415^Val	MW188016	India	*T. indotineae*	0.5	1	1.56
1178T>C	Leu^393^Ser	MW188020	India	*T. indotineae*	0.5	1	1.56
1318C>T	His^440^Tyr	MW187976	India	*T. indotineae*	0.125	1	1.56
1342G>A	Ala^448^Thr	MW187981	India	*T. indotineae*	0.0625–0.25	15	23.4
828G>C, 1255C>T	Lys^276^Asn, Leu^419^Phe	MW188025	China	T. mentagrophytes	0.031	10	

**TABLE 3:** Comparison of AFST results after 10 sequential passages on PDA drug-free medium^*a*^

Parameter	Value (mg/liter)							
	NAF	ITZ	MCZ	KTZ	TBF	SCZ	FCZ	GRI
TBF-resistant species (*n* = 13)								
GM	>16	0.191	1.055	0.474	>16	1.377	24.511	3.409
MIC_50_	>16	0.125	1	0.5	>16	1	32	4
MIC_90_	>16	0.5	2	1	>16	2	>32	4
Range	16 to >16	0.0625 to 1	0.5 to 2	0.25 to 1	>16	1 to 2	16 to >32	2 to 4
Passage 10 times (*n* = 13)								
GM	>16	0.225	1.238	0.653	>16	1.452	28.763	5.222
MIC_50_	>16	0.125	1	0.5	>16	1	32	4
MIC_90_	>16	0.5	2	1	>16	2	>32	4
Range	16 to >16	0.0625 to 1	1 to 2	0.5 to 1	>16	1 to 2	16 to >32	4 to 8

TBF-susceptible species (*n* = 3)								
GM	0.157	0.250	1.260	0.630	0.099	1.587	2.520	2.000
Range	0.0625 to 0.25	0.125 to 0.5	1 to 2	0.25 to 2	0.0625 to 0.125	1 to 2	2 to 4	2.000
Passage 10 times (*n* = 3)								
GM	0.157	0.250	1.000	0.315	0.099	2.000	0.500	3.175
Range	0.031 to 0.5	0.125 to 0.5	1.000	0.25 to 0.5	0.0625 to 0.125	1 to 4	0.500	2 to 4

a The MICs of eight drugs show no statistical difference between 0 and 10 exposures.

### Triazole susceptibility.

**FIG 3:**
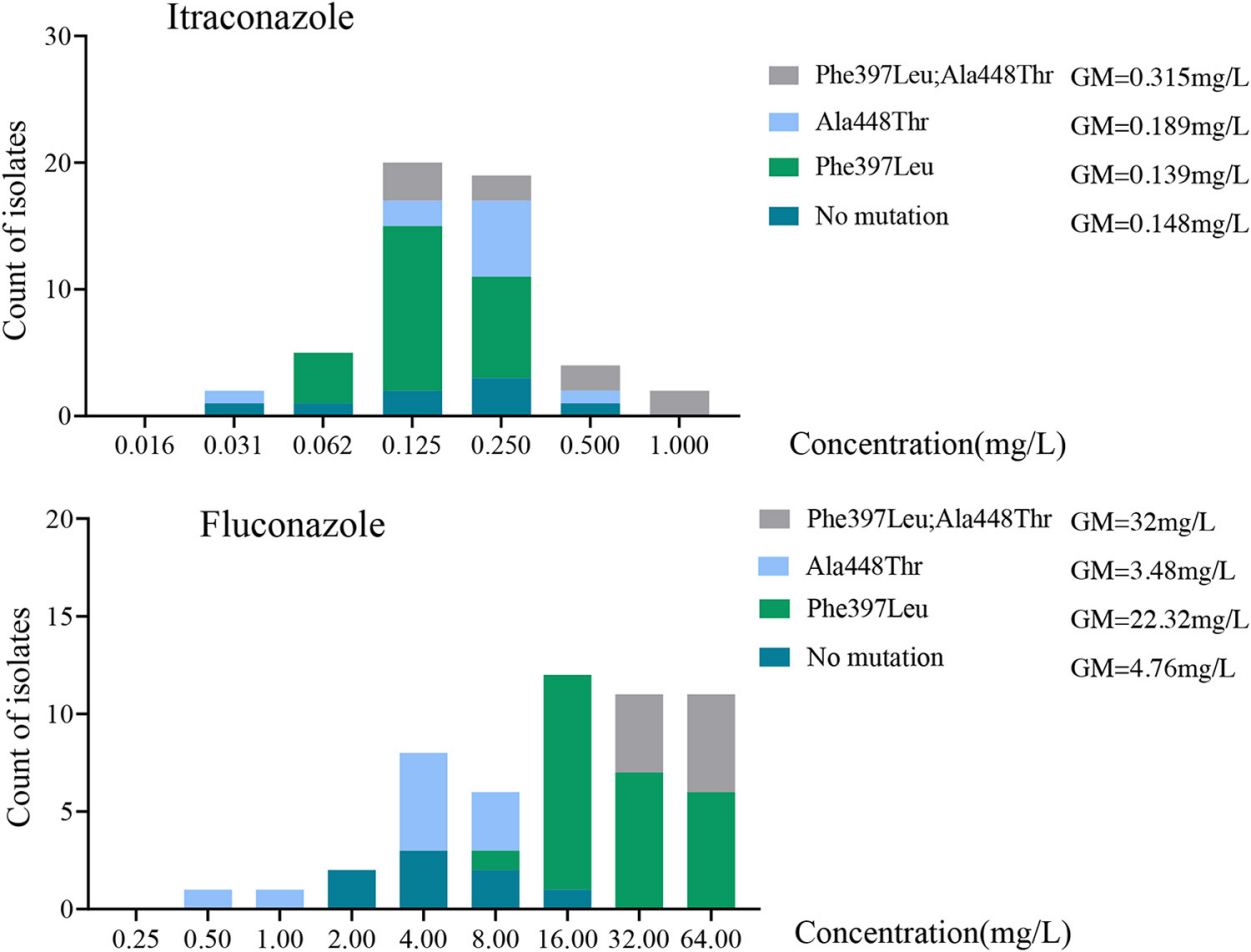
Comparison of amino acid substitutions in the *SQLE* gene influencing triazoles. The double-amino-acid substitution (Phe^397^Leu and Ala^448^Thr) shows higher MIC values of FCZ and ITZ than the single mutations. No statistical difference is observed between the amino acid substitution Ala^448^Thr and no mutation.

MIC values for ITZ and FCZ between *Trichophyton* strains exhibited the four most prevalent *SQLE* genotypes: Phe^397^Leu with concurrent Ala^448^Thr, Ala^448^Thr, Phe^397^Leu, and no mutation. We performed a Kruskal-Wallis test, which yielded significant differences among genotypes for FCZ (*H* = 36.54; *P* < 0.001). The MICs of the triazole FCZ in strains carrying the *SQLE* double substitution Phe^397^Leu/Ala^448^Thr were much higher than those in strains with single Phe^397^Leu or Ala^448^Thr substitutions and no mutation (Fig. 3). For ITZ, there is no statistical difference among genotypes (*P* > 0.05). For FCZ, the GM values in strains with the single Phe^397^Leu mutation were much higher than those in strains with the single Ala^448^Thr mutation or no mutation. This suggests that the Ala^448^Thr mutation is not directly responsible for the increase in resistance against FCZ, with a second, thus-far-unknown factor being needed for the resistant phenotype.

## DISCUSSION

In our experiments, we used dermatophyte strains of the T. mentagrophytes species complex originating from three continents (i.e., China, India, Australia, Germany, and The Netherlands). Strains were grouped into three species, *T. indotineae*, T. interdigitale *sensu stricto*, and T. mentagrophytes *sensu stricto*, based on the latest taxonomic insights (6, 7). Comparing AFST profiles of the 135 strains under study, we noticed that TBF resistance is restricted to *T. indotineae* isolates from India and that these strains also showed cross-resistance to another allylamine drug, NAF. This might support the status of *T. indotineae* as a separate species, showing diagnostic differences in mating type genes and ribosomal DNA (rDNA) ITSs in addition to statistically significant trends in its phenotype (7). The origin of this TBF-resistant species, causing recalcitrant and difficult-to-treat dermatophytosis, which has been emerging in India since 2015, is a much-debated topic (2–4, 6). The inappropriate use of over-the-counter creams with combined steroid and antifungal mixtures is suggested to be the probable cause of the spread of this problematic species (1).

Comparison with published data is hampered by taxonomic confusion concerning the T. mentagrophytes species complex. While recent data have shown that the correct name of the emerging species in India is *T. indotineae*, it has been variably identified as T. mentagrophytes genotype VIII or T. interdigitale, but correction as *T. indotineae* is possible via sequences deposited in GenBank. In strains retrospectively identified as belonging to the T. mentagrophytes species complex, a significant spread of TBF resistance ranging between 0.2 and 81% is observed (Table 4). Although no clinical breakpoints (CBPs) for MIC values have been established to guide antifungal therapy, epidemiological cutoff (ECV or ECOFF) values may serve as a substitute for the detection of resistance. The ECOFF is the highest MIC value for isolates devoid of phenotypically detectable resistance mechanisms. As a first step, the ECOFF needs to be based on the new taxonomy. Shaw et al. (16) suggested that determination of the UL-WT (upper limit of the wild type) may be beneficial for managing dermatophytosis and monitoring the emergence of isolates with reduced susceptibility. The MICs comprising >95% of the modeled populations were 8 mg/liter for TBF and 32 mg/liter for FCZ. Arendrup et al. (17, 18) reported that TBF resistance can be set at 0.25 mg/liter (visually) or 0.125 mg/liter (spectrophotometrically at the 50% inhibition endpoint). These ECOFFs may be helpful for physicians in the current scenario of recalcitrant dermatophytes. In our data, *T. indotineae* shows an almost bimodal distribution (Fig. 1) with isolates having an MIC of >0.25 mg/liter (visually). A value of 0.25 mg/liter was found, in consensus with the UL-WT calculating result, for determinations visually and spectrophotometrically at the 90% endpoint according to the EUCAST method (16). Thus, a uniform standard for the definition of TBF resistance is required. Due to the low number of available strains of *Trichophyton* species, an ECOFF could not be calculated in our experiments. Based on the UL-WT according to Arendrup et al. (18), the rate of resistance to TBF in our experiments was 58% (upper limit of the wild type set at an MIC of ≥0.25 mg/liter). In accordance with other experiments (19), strains with an MIC of TBF of ≥0.25 mg/liter are able to survive on RPMI 1640 agar containing 0.2 mg/liter terbinafine. We consequently used this method to screen for strains with increased resistance to TBF.

**TABLE 4:** Number of TBF-resistant isolates showing different countries and mutations in the *SQLE* gene

Reference	Country	No. of all isolates	No. of resistant isolates	MIC (mg/liter)	Resistance rate (%)	Mutation(s)
10	Switzerland	412	1	3.2	0.24	Phe^397^Leu
36	India	64	39	1 to ≥32	60.94	Phe^397^Leu, Leu^393^Phe
2	India	63	20	4 to ≥32	31.75	Leu^393^Phe, Phe^397^Leu
5	India	129	105	1 to ≥32	81.40	Leu^393^Phe, Phe^397^Leu
37	Japan	24	1	2	4.17	None
24	India	146	15	≥2	10.27	Phe^397^Leu
16	India	498	102	1 to ≥32	20.50	Phe^397^Leu, Leu^393^Phe
19	India	279	202	0.25	72.00	Leu^393^Ser, Gln^408^Leu, His^440^Tyr, Ser^443^Pro, Leu^335^Phe, Ser^395^Pro
12	Iran	141	5	≥32	3.54	Phe^397^Leu, Leu^393^Ser

In order to classify the background of resistance in *T. indotineae*, we selected all resistant strains and 15 susceptible strains of this species and 10 susceptible isolates each of T. mentagrophytes and T. interdigitale. These were used for the detection of mutations in the *SQLE* gene. In accordance with previous reports (2, 12), a high MIC (≥16 mg/liter) (53.13%) of TBF isolates was associated with mutation 1189T>C, 1191C>A, or 1191C>G, which led to the amino acid substitution Phe^397^Leu; sometimes, this mutation was combined with the amino acid substitution Ala^448^Thr. Strains with moderate to low MICs for TBF (0.125 to 0.5 mg/liter) showed the amino acid substitutions Phe^415^Val, Leu^393^Ser, His^440^Tyr, and Ala^448^Thr (11, 12). Unexpectedly, several susceptible strains (MICs of 0.016 to 0.125 mg/liter) of *T. indotineae* had the mutation 1342G>A (Ala^448^Thr). A search conducted in GenBank revealed sequences (accession numbers MN893286 and MN901902 to MN901905) of three susceptible strains with the same mutation (1342G>A). Consequently, it was concluded that this mutation alone does not contribute to TBF resistance. It may be a silent mutation or perhaps may contribute to the increased MIC values observed for triazoles. The role of this amino acid substitution remains as yet unknown. Isolates from China and Germany identified as T. mentagrophytes carried the thus-far-unreported mutations 828G>C and 1255C>T, leading to Lys^276^Asn and Leu^419^Phe, respectively, and may be unique in T. mentagrophytes. But due to the limitation of detected strains, only 10 strains each of T. mentagrophytes and T. interdigitale were sequenced. Possibly, this parameter adds to the distinction of the closely related species T. mentagrophytes and T. interdigitale, which needs more data to demonstrate.

Amino acid substitutions variably impact conformational changes in the active site of the enzyme resulting in reduced drug affinity. Leber et al. (20) generated TBF-resistant clones of Saccharomyces cerevisiae by mutagenesis. Subsequent molecular analysis of the *SQLE* gene revealed point mutations leading to amino acid substitutions at Phe402, Phe420, and Phe430, respectively (21). The residues Phe402 and Phe420 in S. cerevisiae *SQLE* correspond to Phe397 and Phe415 in *T. indotineae* SQLE, respectively. This means that this mutation is highly conserved over large phylogenetic distances. Nowosielski et al. (22) established that these regions were localized to the C terminus of the *SQLE* gene and speculated that the amino acid changes influence the interaction with the drug. Homology modeling was performed to compare the mutated residue and the wild type, showing that the mutant residue is smaller than the original, interfering with the binding of the molecule and resulting in a failure of drug-enzyme interactions (23). Shankarnarayan et al. (24) put forward that *SQLE* could serve as a marker of resistance, especially the substitutions between Leu393 and Ser443 (16). Our data show that the three species in the T. mentagrophytes complex each harbor specific mutations in the *SQLE* gene.

Triazoles specifically interact with the lanosterol 14-alpha demethylase (*ERG11*) gene, influencing the synthesis of ergosterol. Hence, *ERG1* mutants lacking ergosterol are expected to be resistant to these drugs. However, some reports showed that an *ERG1* mutant led to increased susceptibility to drugs in *Candida* (25, 26); thus, the exact mechanism of *ERG1* influencing *ERG11* is not clear in *T. indotineae*.

In our data set, the MICs of FCZ proved to be much higher in TBF-resistant strains of *T. indotineae* than in T. mentagrophytes *sensu stricto* and T. interdigitale *sensu stricto*. Strains with the double mutations of the *SQLE* gene that cause the amino acid substitutions Phe^397^Leu and Ala^448^Thr showed increased MIC values of FCZ, which was not observed in strains with a single mutation. Burmester et al. (27) speculated that the Ala^448^Thr substitution combined with the Phe^397^Leu substitution in the *SQLE* gene may contribute to higher FCZ and VCZ MICs; however, the Gln^408^Leu and Ala^448^Leu double mutants did not follow this trend. In our analysis, no statistical difference was found between the single mutation Ala^448^Thr and no mutation. With FCZ, a statistically significant association is observed between Phe^397^Leu and no mutation. We therefore speculated that Ala^448^Thr alone might not lead to higher FCZ and ITZ MICs, but combined with Phe^397^Leu, an effect on FCZ and ITZ can be observed, explaining the significantly higher MICs of FCZ in *T. indotineae*. Similar observations have been reported previously by Ebert et al. (19). The amino acid substitution Ala^448^Thr at the C terminus of squalene epoxidase probably has an impact on subsequent steps in ergosterol synthesis through conformational changes and may result in reduced susceptibility to azoles. Besides, Gnat et al. (28) indicated higher levels of *PDR1*, *MDR2*, and *MDR4* gene expression in terbinafine-resistant T. mentagrophytes isolates after exposure to a subinhibitory concentration of terbinafine than in the case of cells incubated without the antifungal. Kano et al. (29) revealed that the expression levels of the *PDR1*, *MDR1*, *MDR2*, and *MDR4* genes were 2 to 4 times higher in the TBF-resistant strain grown in the presence of 0.14 μg/ml of TBF than in TBF-susceptible strains cultured in the absence of TBF. The conclusions from previous studies that disruption of the *MDR2* gene renders mutants more susceptible to terbinafine than control strains suggest that one alternative to *SQLE* point mutation mechanisms of terbinafine resistance may involve efflux pumps (30).

In conclusion, by using the new taxonomy of the T. mentagrophytes species complex, we describe the MIC distributions of eight drugs. High MICs of terbinafine, with the amino acid substitution Phe^397^Leu, are as yet limited to *T. indotineae* and are unlikely to be distributed throughout the T. mentagrophytes species complex by genetic exchange.

## MATERIALS AND METHODS

### Clinical specimens and fungal isolates.

A total of 135 clinical isolates from India (*n* = 64), China (*n* = 36), Australia (*n* = 12), Germany (*n* = 7), and The Netherlands (*n* = 16) collected over a 2-year period (2018 to 2019) were used and are shown in Table S1 in the supplemental material. The isolates originated from human cases of tinea corporis, tinea cruris, tinea faciei, or tinea unguium. Skin scrapings and nail clippings were processed for direct microscopic examination with 10% potassium hydroxide (KOH) (31) or an optical brightener. Strains were maintained in Sabouraud’s glucose broth (SGB) containing glycerol and dimethyl sulfoxide (DMSO) at −80°C (32). Microconidium formation was enhanced by culturing on potato dextrose agar (PDA; Oxoid, Basingstoke, England) at 28°C for 7 to 14 days.

### Identification.

For DNA extraction, mycelium was harvested from 7- to 14-day-old cultures grown on PDA at 28°C. Mycelial fragments (approximately 0.5 cm in diameter) were soaked in breaking buffer (2% Triton X-100, 1% sodium dodecyl sulfate [SDS], 100 mM NaCl, 10 mM Tris HCl, 1 mM EDTA [pH 8]) with 300 mg glass beads (0.4 to 0.6 mm in diameter) (2, 12). Samples were shaken at 1,200 rpm at 70°C for 30 min, 250 μl phenol-chloroform-isoamyl alcohol (Invitrogen, St. Louis, MO, USA) was added, and the mixture was shaken again for 5 min at room temperature, followed by centrifugation at 11,000 × *g* for 5 min. The top fraction was collected and stored at −20°C. Species identification was done using the rDNA internal transcribed spacer (ITS) with primers ITS-1 (5′-TCCGTAGGTGAACCTTGCGG-3′) and ITS-4 (5′-TCCTCCGCTTATTGATATGC-3′) (33). Diagnostic high-mobility-group (HMG) data were provided by C. Tang (7). PCR products were purified using gel electrophoresis (QIAquick; Qiagen, Hilden, Germany) and sequenced using an ABI BigDye Terminator (v3.1) cycle sequencing kit. The sequencing reactions were done at 95°C for 1 min with 30 cycles of 95°C for 10 s, 50°C for 5 s, and 60°C for 4 min on an ABI 3730XL automatic sequencer (Applied Biosystems, Foster City, CA, USA) with the ABI Prism BigDye Terminator cycle sequencing kit (Applied Biosystems). Sequences were aligned using CodonCode Aligner software (CodonCode Corp., Centerville, MA, USA) and compared to data in GenBank.

### Antifungal susceptibility testing.

Microtiter plates (Costar; Corning, NY, USA) were prepared using 2-fold serial dilutions in double-concentrated medium according to the EUCAST E.Def 9.3.1 protocol (12, 34). Strains were subcultured on PDA for 7 to 14 days at 28°C, and conidial inocula were prepared in MilliQ water containing Tween 20 (0.5%) by gently scraping the surface of mature colonies with a sterile cotton swab and using filtration on a nylon filter with a porosity of 11 μm. An initial inoculum corresponding to 80 to 83% transmittance at 530 nm in a spectrophotometer was used and diluted 1:10 with sterile water to obtain final inocula of 0.5 × 10^5^ to 2.5 × 10^5^ CFU/ml; concentrations were determined again using a hemocytometer. Antifungals were purchased from Sigma-Aldrich and dissolved in DMSO (5 mg/liter; Sigma-Aldrich, Burlington, MA, USA). Stock solutions were prepared at concentrations of 3,200 mg/liter for all drugs. The applied concentration ranges were 0.016 to 16 mg/liter for itraconazole (ITZ), ketoconazole (KTZ), miconazole (MCZ), naftifine (NAF), sertaconazole (SCZ), and terbinafine (TBF) and 0.03 to 32 mg/liter for fluconazole (FCZ) and griseofulvin (GRI). Microtiter plates were frozen at −80°C for at least 24 h prior to use. Reference strains T. interdigitale ATCC MYA-4439, Candida krusei ATCC 6258, and Candida parapsilosis ATCC 22019 were used as controls and were read after 24 h of incubation (for yeasts) at 37°C. Drug-free controls were included, and microtiter plates were incubated at 28°C (for filamentous fungi). MIC endpoints for all drugs were defined as the lowest concentration that produced complete inhibition of growth as read visually after 5 days; for yeasts, results are read at 50% inhibition by using a spectrophotometer.

### Screening for TBF and ITZ resistance.

In order to validate a previously described (10) agar method for detecting TBF resistance in *Trichophyton*, we screened 50 isolates (MIC of TBF of ≥16 mg/liter, *n* = 34; MIC of TBF of 0.125 to 0.5 mg/liter, *n* = 10; MIC of TBF of 0.016 to 0.062 mg/liter, *n* = 6; MIC of ITZ of 0.062 to 2 mg/liter, all isolates) on RPMI 1640 agar plates containing 0.2 mg/liter TBF and 4 mg/liter ITZ (35). RPMI 1640 medium (Biowest, Nuaillé, France) supplemented with 2% glucose (Sigma, St. Louis, MO, USA) and 0.165 mol/liter 3-*N*-morpholinepropanesulfonic acid (MOPS) (pH 7.0) (Sigma) was mixed with double-strength concentrations of Bacto agar (Becton, Dickinson, Sparks, MD, USA) and subsequently added to the working solutions of each compound, and the mixture was autoclaved at 121°C for 15 min. The antifungal-free mixture was used in the control wells. Prepared RPMI 1640 agar was applied to 24-well plates with 1.5 ml/well and stored at 4°C. The concentration of TBF was qualitatively equivalent to twice the previously described MIC for *Trichophyton* species (14). The cultured colonies were then inoculated at the center of the RPMI 1640 agar plates containing TBF or ITZ and incubated at 28°C. Fungal growth ability was examined after 5, 7, and 14 days.

### Squalene epoxidase gene sequencing.

A total of 62 isolates were screened for mutations in the squalene epoxidase (*SQLE*) gene. The primers used are listed in Table 5. PCR was carried out in 50-μl reaction mixture volumes, and conditions included an initial denaturation step for 5 min at 94°C followed by 35 cycles of 30 s at 94°C, 45 s at 59°C, and 100 s at 72°C. PCR products were purified using gel electrophoresis (QIAquick) and sequenced using the primers listed in Table 5. DNA sequencing was performed with PCR primers at a concentration of 2.5 mmol/liter. Gene and amino acid sequences of *SQLE* of *Trichophyton* spp. were compared with those of T. interdigitale (GenBank accession numbers KK201110.1 and EZF33561).

**TABLE 5:** PCR primers used in this study

Purpose	Primer	Sequence (5′–3′)
ITS1	ITS1	TCCGTAGGTGAACCTGCGG

ITS4	ITS4	TCCTCCGCTTATTGATATGC

*SQLE* amplification	SQLE-fw1	AGCTGGCAGACTTCCTTTATC
	SQLE-rv1	GCAGAGATAATGCAGCCACC

*SQLE* sequencing	SQLE-fw1	AGCTGGCAGACTTCCTTTATC
	SQLE-fw2	GTCACCATTGTCGAGACCAAG
	SQLE-fw3	GATTGATGTTCCTAGGTGACT
	SQLE-rv1	TTAAATGCCACGGTCATACCG
	SQLE-rv2	CTTTCGGAACGTAGAGGCATA
	SQLE-rv3	GCAGAGATAATGCAGCCACC

### Stability of TBF resistance.

Sixteen isolates (TBF MIC of ≥16 mg/liter, *n* = 13; TBF MIC of 0.016 to 0.062 mg/liter, *n* = 3) were subjected to sequential passage 10 times on drug-free Sabouraud dextrose agar (SDA) at 7- to 10-day intervals. Subsequently, the 20 strains and control strains were analyzed by AFST (FCZ, GRI, ITZ, KTZ, MCZ, NAF, SCZ, and TBF) according to the methods described above.

### Statistics.

Figures were created by using GraphPad Prism version 8.0.0 (GraphPad Software), and Kruskal-Wallis tests were performed using SPSS version 26 (IBM); differences were considered statistically significant at *P* values of ≤0.05.

### Data availability.

The ITS sequences of newly sequenced strains were deposited in GenBank under accession numbers MW346048 to MW346178. The *SQLE* sequences of newly sequenced strains were deposited in GenBank under accession numbers MW187971 to MW188029.1.
