# Supplementary material for: Antifungal Susceptibility and Mutations in the Squalene Epoxidase Gene in Dermatophytes of the Trichophyton mentagrophytes Species Complex
Source: Antimicrob Agents Chemother. 2021 Jul 16;65(8):e00056-21. doi: 10.1128/AAC.00056-21 (PMC8284460; doi:10.1128/AAC.00056-21)
Supplement: Supplemental file 1 — Supplemental Tables S1 and S2. Download AAC00056-21_Supp_1_seq12.pdf, PDF file, 0.1 MB [file aac00056-21_supp_1_seq12.pdf]

Table S-1. Metadata of strains used in the study.

Number	Original Number	Country	Source	Collect by	ITS Genotype	multilocus comparison (ITS,*Tef1-α,HMG*)
1	XM2	China	Tinea faciei	Ping Zhan	*T. interdigitale*	*T. interdigitale*
2	XM9	China	Tinea faciei	Ping Zhan	*T. interdigitale*	*T. interdigitale*
3	XM10	China	Onychomycosis	Ping Zhan	*T. interdigitale*	*T. interdigitale*
4	XM16	China	Tinea faciei	Ping Zhan	*T. interdigitale*	*T. interdigitale*
5	XM30	China	Tinea faciei	Ping Zhan	*T. interdigitale*	*T. interdigitale*
6	XM32	China	Tinea capitis	Ping Zhan	*T. interdigitale*	*T. interdigitale*
7	XM40	China	Tinea corporis	Ping Zhan	*T. interdigitale*	*T. interdigitale*
8	XM45	China	Tinea faciei	Ping Zhan	*T. interdigitale*	*T. interdigitale*
9	XM48	China	Tinea capitis	Ping Zhan	*T. interdigitale*	*T. interdigitale*
10	208	Netherlands	Tinea	Hein van der Lee	*T. interdigitale*	*T. interdigitale*
11	216	Netherlands	Tinea	Hein van der Lee	*T. interdigitale*	*T. interdigitale*
12	276	Netherlands	Tinea	Hein van der Lee	*T. interdigitale*	*T. interdigitale*
13	278	Netherlands	Tinea	Hein van der Lee	*T. interdigitale*	*T. interdigitale*
14	321	Netherlands	Tinea	Hein van der Lee	*T. interdigitale*	*T. interdigitale*
15	335	Netherlands	Tinea	Hein van der Lee	*T. interdigitale*	*T. interdigitale*
16	394	Netherlands	Tinea	Hein van der Lee	*T. interdigitale*	*T. interdigitale*
17	421	Netherlands	Tinea	Hein van der Lee	*T. interdigitale*	*T. interdigitale*
18	439	Netherlands	Tinea	Hein van der Lee	*T. interdigitale*	*T. interdigitale*
19	440	Netherlands	Tinea	Hein van der Lee	*T. interdigitale*	*T. interdigitale*
20	V10-04	Netherlands	unknown	Hein van der Lee	*T. interdigitale*	*T. interdigitale*
21	V10-41	Netherlands	Tinea	Hein van der Lee	*T. interdigitale*	*T. interdigitale*
22	V21-14	Netherlands	Onychomycosis	Hein van der Lee	*T. interdigitale*	*T. interdigitale*
23	V70-35	Netherlands	Tinea	Hein van der Lee	*T. interdigitale*	*T. interdigitale*
24	V155-6	Netherlands	unknown	Hein van der Lee	*T. interdigitale*	*T. interdigitale*
25	A11	Australia	Onychomycosis	Steven	*T. interdigitale*	*T. interdigitale*
26	A18	Australia	Onychomycosis	Steven	*T. interdigitale*	*T. interdigitale*
27	A31	Australia	Onychomycosis	Steven	*T. interdigitale*	*T. interdigitale*
28	A44	Australia	Onychomycosis	Steven	*T. interdigitale*	*T. interdigitale*
29	A50	Australia	Onychomycosis	Steven	*T. interdigitale*	*T. interdigitale*
30	A60	Australia	Onychomycosis	Steven	*T. interdigitale*	*T. interdigitale*
31	A73	Australia	Onychomycosis	Steven	*T. interdigitale*	*T. interdigitale*
32	A171	Australia	Onychomycosis	Steven	*T. interdigitale*	*T. interdigitale*
33	A177	Australia	Onychomycosis	Steven	*T. interdigitale*	*T. interdigitale*
34	A191	Australia	Onychomycosis	Steven	*T. interdigitale*	*T. interdigitale*
35	A221	Australia	Onychomycosis	Steven	*T. interdigitale*	*T. interdigitale*
36	A238	Australia	Onychomycosis	Steven	*T. interdigitale*	*T. interdigitale*
37	218893/16	Germany	Tinea capitis	Nenoff	*T. mentagrophytes III**	*T. mentagrophytes*
38	200602/17	Germany	Tinea corporis	Nenoff	*T. mentagrophytes IV*	*T. mentagrophytes*
39	200617/17	Germany	Tinea corporis	Nenoff	*T. mentagrophytes IV*	*T. mentagrophytes*
40	204543/17	Germany	Tinea	Nenoff	*T. mentagrophytes IV*	*T. mentagrophytes*
41	V155-32	Netherlands	unknown	Hein van der Lee	*T. mentagrophytes IV*	*T. mentagrophytes*

42	XM20	China	Tinea capitis	Ping Zhan	*T. mentagrophytes IV*	*T. mentagrophytes*
43	XM21	China	Tinea capitis	Ping Zhan	*T. mentagrophytes IV*	*T. mentagrophytes*
44	210363/16	Germany	Tinea genitalis	Nenoff	*T. mentagrophytes VII*	*T. mentagrophytes*
45	218904/16	Germany	Tinea genitalis	Nenoff	*T. mentagrophytes VII*	*T. mentagrophytes*
46	215003/16	Germany	Tinea genitalis	Nenoff	*T. mentagrophytes VII*	*T. mentagrophytes*
47	XM1	China	Tinea ear	Ping Zhan	*T. mentagrophytes IX*	*T. mentagrophytes*
48	XM4	China	Tinea capitis	Ping Zhan	*T. mentagrophytes IX*	*T. mentagrophytes*
49	XM5	China	Tinea faciei	Ping Zhan	*T. mentagrophytes IX*	*T. mentagrophytes*
50	XM6	China	Tinea faciei	Ping Zhan	*T. mentagrophytes IX*	*T. mentagrophytes*
51	XM7	China	Tinea cruris	Ping Zhan	*T. mentagrophytes IX*	*T. mentagrophytes*
52	XM8	China	Tinea capitis	Ping Zhan	*T. mentagrophytes IX*	*T. mentagrophytes*
53	XM11	China	Tinea faciei	Ping Zhan	*T. mentagrophytes IX*	*T. mentagrophytes*
54	XM12	China	Tinea faciei	Ping Zhan	*T. mentagrophytes IX*	*T. mentagrophytes*
55	XM14	China	Tinea capitis	Ping Zhan	*T. mentagrophytes IX*	*T. mentagrophytes*
56	XM19	China	Tinea capitis	Ping Zhan	*T. mentagrophytes IX*	*T. mentagrophytes*
57	XM22	China	Tinea faciei	Ping Zhan	*T. mentagrophytes IX*	*T. mentagrophytes*
58	XM26	China	Tinea capitis	Ping Zhan	*T. mentagrophytes IX*	*T. mentagrophytes*
59	XM27	China	Tinea capitis	Ping Zhan	*T. mentagrophytes IX*	*T. mentagrophytes*
60	XM34	China	Tinea corporis	Ping Zhan	*T. mentagrophytes IX*	*T. mentagrophytes*
61	XM35	China	Tinea capitis	Ping Zhan	*T. mentagrophytes IX*	*T. mentagrophytes*
62	XM36	China	Tinea capitis	Ping Zhan	*T. mentagrophytes IX*	*T. mentagrophytes*
63	XM37	China	Tinea capitis	Ping Zhan	*T. mentagrophytes IX*	*T. mentagrophytes*
64	XM39	China	Tinea capitis	Ping Zhan	*T. mentagrophytes IX*	*T. mentagrophytes*
65	XM41	China	Uknown	Ping Zhan	*T. mentagrophytes IX*	*T. mentagrophytes*
66	XM42	China	Tinea faciei	Ping Zhan	*T. mentagrophytes IX*	*T. mentagrophytes*
67	XM46	China	Tinea faciei	Ping Zhan	*T. mentagrophytes IX*	*T. mentagrophytes*
68	XM49	China	Tinea faciei	Ping Zhan	*T. mentagrophytes IX*	*T. mentagrophytes*
69	XM54	China	Uknown	Ping Zhan	*T. mentagrophytes IX*	*T. mentagrophytes*
70	XM55	China	Tinea cruris	Ping Zhan	*T. mentagrophytes IX*	*T. mentagrophytes*
71	XM56	China	Uknown	Ping Zhan	*T. mentagrophytes IX*	*T. mentagrophytes*
72	211497/17	West India	Tinea corporis	Nenoff	*T. mentagrophytes VIII*	*T. indotineae*
73	211509/17	West India	Tinea	Nenoff	*T. mentagrophytes VIII*	*T. indotineae*
74	216500/17	Nord India	Tinea	Nenoff	*T. mentagrophytes VIII*	*T. indotineae*
75	216520/17	Nord India	Tinea	Nenoff	*T. mentagrophytes VIII*	*T. indotineae*
76	200074/18	South India	Tinea	Nenoff	*T. mentagrophytes VIII*	*T. indotineae*
77	200095/18	South India	Tinea	Nenoff	*T. mentagrophytes VIII*	*T. indotineae*
78	200100/18	West India	Tinea corporis	Nenoff	*T. mentagrophytes VIII*	*T. indotineae*
79	200101/18	West India	Tinea corporis	Nenoff	*T. mentagrophytes VIII*	*T. indotineae*
80	1728	East India	Tinea	Nenoff	*T. mentagrophytes VIII*	*T. indotineae*
81	i2	India	Tinea cruris	Rameshwari	*T. mentagrophytes VIII*	*T. indotineae*
82	i3	India	Tinea cruris	Rameshwari	*T. mentagrophytes VIII*	*T. indotineae*
83	i5	India	Tinea manuum	Rameshwari	*T. mentagrophytes VIII*	*T. indotineae*
84	i7	India	Tinea cruris	Rameshwari	*T. mentagrophytes VIII*	*T. indotineae*
85	i8	India	Tinea cruris	Rameshwari	*T. mentagrophytes VIII*	*T. indotineae*

86	i10	India	Tinea cruris	Rameshwari	*T. mentagrophytes VIII*	*T. indotineae*
87	i11	India	Tinea pedis	Rameshwari	*T. mentagrophytes VIII*	*T. indotineae*
88	i12	India	Tinea cruris	Rameshwari	*T. mentagrophytes VIII*	*T. indotineae*
89	i14	India	Tinea cruris	Rameshwari	*T. mentagrophytes VIII*	*T. indotineae*
90	i15	India	Tinea cruris	Rameshwari	*T. mentagrophytes VIII*	*T. indotineae*
91	i16	India	Tinea faciei	Rameshwari	*T. mentagrophytes VIII*	*T. indotineae*
92	i19	India	Tinea cruris	Rameshwari	*T. mentagrophytes VIII*	*T. indotineae*
93	i20	India	Tinea cruris	Rameshwari	*T. mentagrophytes VIII*	*T. indotineae*
94	i21	India	Tinea cruris	Rameshwari	*T. mentagrophytes VIII*	*T. indotineae*
95	i23	India	Tinea cruris	Rameshwari	*T. mentagrophytes VIII*	*T. indotineae*
96	i24	India	Tinea cruris	Rameshwari	*T. mentagrophytes VIII*	*T. indotineae*
97	i25	India	Tinea cruris	Rameshwari	*T. mentagrophytes VIII*	*T. indotineae*
98	i26	India	Tinea cruris	Rameshwari	*T. mentagrophytes VIII*	*T. indotineae*
99	i27	India	Tinea cruris	Rameshwari	*T. mentagrophytes VIII*	*T. indotineae*
100	i29	India	Tinea cruris	Rameshwari	*T. mentagrophytes VIII*	*T. indotineae*
101	i32	India	Tinea cruris	Rameshwari	*T. mentagrophytes VIII*	*T. indotineae*
102	i35	India	Tinea cruris	Rameshwari	*T. mentagrophytes VIII*	*T. indotineae*
103	i36	India	Tinea cruris	Rameshwari	*T. mentagrophytes VIII*	*T. indotineae*
104	i38	India	Tinea cruris	Rameshwari	*T. mentagrophytes VIII*	*T. indotineae*
105	i39	India	Tinea cruris	Rameshwari	*T. mentagrophytes VIII*	*T. indotineae*
106	i40	India	Tinea cruris	Rameshwari	*T. mentagrophytes VIII*	*T. indotineae*
107	i41	India	Tinea cruris	Rameshwari	*T. mentagrophytes VIII*	*T. indotineae*
108	i42	India	Tinea cruris	Rameshwari	*T. mentagrophytes VIII*	*T. indotineae*
109	i43	India	Tinea cruris	Rameshwari	*T. mentagrophytes VIII*	*T. indotineae*
110	i47	India	Tinea cruris	Rameshwari	*T. mentagrophytes VIII*	*T. indotineae*
111	i48	India	Tinea cruris	Rameshwari	*T. mentagrophytes VIII*	*T. indotineae*
112	i49	India	Tinea faciei	Rameshwari	*T. mentagrophytes VIII*	*T. indotineae*
113	V245-44	India	Unknown	Ashutosh Singh	*T. mentagrophytes VIII*	*T. indotineae*
114	V245-45	India	Unknown	Ashutosh Singh	*T. mentagrophytes VIII*	*T. indotineae*
115	V245-46	India	Unknown	Ashutosh Singh	*T. mentagrophytes VIII*	*T. indotineae*
116	V245-50	India	Unknown	Ashutosh Singh	*T. mentagrophytes VIII*	*T. indotineae*
117	V245-54	India	Unknown	Ashutosh Singh	*T. mentagrophytes VIII*	*T. indotineae*
118	V245-81	India	Unknown	Ashutosh Singh	*T. mentagrophytes VIII*	*T. indotineae*
119	V246-01	India	Unknown	Ashutosh Singh	*T. mentagrophytes VIII*	*T. indotineae*
120	V246-03	India	Unknown	Ashutosh Singh	*T. mentagrophytes VIII*	*T. indotineae*
121	V246-04	India	Unknown	Ashutosh Singh	*T. mentagrophytes VIII*	*T. indotineae*
122	V246-05	India	Unknown	Ashutosh Singh	*T. mentagrophytes VIII*	*T. indotineae*
123	V246-08	India	Unknown	Ashutosh Singh	*T. mentagrophytes VIII*	*T. indotineae*
124	V246-11	India	Unknown	Ashutosh Singh	*T. mentagrophytes VIII*	*T. indotineae*
125	V246-12	India	Unknown	Ashutosh Singh	*T. mentagrophytes VIII*	*T. indotineae*
126	V246-13	India	Unknown	Ashutosh Singh	*T. mentagrophytes VIII*	*T. indotineae*
127	V246-14	India	Unknown	Ashutosh Singh	*T. mentagrophytes VIII*	*T. indotineae*
128	V246-16	India	Unknown	Ashutosh Singh	*T. mentagrophytes VIII*	*T. indotineae*
129	V246-19	India	Unknown	Ashutosh Singh	*T. mentagrophytes VIII*	*T. indotineae*

130	V246-20	India	Unknown	Ashutosh Singh	*T. mentagrophytes VIII*	*T. indotineae*
131	V246-21	India	Unknown	Ashutosh Singh	*T. mentagrophytes VIII*	*T. indotineae*
132	V246-22	India	Unknown	Ashutosh Singh	*T. mentagrophytes VIII*	*T. indotineae*
133	V246-23	India	Unknown	Ashutosh Singh	*T. mentagrophytes VIII*	*T. indotineae*
134	V246-24	India	Unknown	Ashutosh Singh	*T. mentagrophytes VIII*	*T. indotineae*
135	V246-25	India	Unknown	Ashutosh Singh	*T. mentagrophytes VIII*	*T. indotineae*

Table S2. The result of strains (n=50) grown on containing TBF and ITZ agar.

Number	Original Number	multilocus comparison (ITS, *Tef1-α*, *HMG*)	MIC of TBF (mg/l)	RPMI 1640 agar TBF (0.2mg/l)	MIC of ITZ (mg/l)	RPMI 1640 agar ITZ (4.0mg/l)
1	i2	T. indotineae	>16	Positive	0.125	Negative
2	i5	T. indotineae	0.25	Positive	0.5	Negative
3	i7	T. indotineae	>16	Positive	0.25	Negative
4	i8	T. indotineae	>16	Positive	0.125	Negative
5	i11	T. indotineae	>16	Positive	0.125	Negative
6	i14	T. indotineae	>16	Positive	1.0	Negative
7	i15	T. indotineae	>16	Positive	0.125	Negative
8	i19	T. indotineae	>16	Positive	0.0625	Negative
9	i21	T. indotineae	>16	Positive	0.125	Negative
10	i24	T. indotineae	>16	Positive	0.25	Negative
11	i25	T. indotineae	>16	Positive	0.25	Negative
12	i26	T. indotineae	>16	Positive	0.125	Negative
13	i32	T. indotineae	>16	Positive	1.0	Negative
14	i35	T. indotineae	>16	Positive	0.125	Negative
15	i36	T. indotineae	>16	Positive	0.25	Negative
16	i38	T. indotineae	>16	Positive	0.25	Negative
17	i39	T. indotineae	>16	Positive	0.0625	Negative
18	i40	T. indotineae	>16	Positive	1.0	Negative
19	i42	T. indotineae	>16	Positive	0.25	Negative
20	i43	T. indotineae	>16	Positive	0.125	Negative
21	i48	T. indotineae	>16	Positive	0.125	Negative
22	i49	T. indotineae	>16	Positive	0.125	Negative
23	V245-45	T. indotineae	>16	Positive	0.125	Negative
24	V245-50	T. indotineae	>16	Positive	0.125	Negative
25	V245-81	T. indotineae	>16	Positive	0.25	Negative
26	V246-01	T. indotineae	>16	Positive	0.125	Negative
27	V246-03	T. indotineae	>16	Positive	0.0625	Negative
28	V246-04	T. indotineae	>16	Positive	0.25	Negative
29	V246-13	T. indotineae	0.5	Positive	0.031	Negative
30	V246-14	T. indotineae	>16	Positive	0.5	Negative
31	V246-16	T. indotineae	>16	Positive	0.125	Negative

32	V246-20	T. indotineae	0.5	Positive	0.031	Negative
33	V246-25	T. indotineae	>16	Positive	0.125	Negative
34	211497/17	T. indotineae	>16	Positive	0.25	Negative
35	200074/18	T. indotineae	>16	Positive	0.25	Negative
36	200095/18	T. indotineae	>16	Positive	0.125	Negative
37	200100/18	T. indotineae	>16	Positive	0.625	Negative
38	ATCC MYA-4439	T. interdigitale	0.016	Negative	0.125	Negative
39	211509/17	T. indotineae	0.125	Negative	0.125	Negative
40	i12	T. indotineae	0.125	Negative	0.25	Negative
41	i10	T. indotineae	0.125	Negative	0.25	Negative
42	i16	T. indotineae	0.0625	Negative	0.125	Negative
43	i20	T. indotineae	0.0625	Negative	0.125	Negative
44	i23	T. indotineae	0.125	Negative	0.25	Negative
45	i27	T. indotineae	0.0625	Negative	0.031	Negative
46	i41	T. indotineae	0.0625	Negative	0.25	Negative
47	i47	T. indotineae	0.0625	Negative	0.25	Negative
48	V245-46	T. indotineae	0.0625	Negative	0.0625	Negative
49	V246-11	T. indotineae	0.125	Negative	0.25	Negative
50	V246-12	T. indotineae	0.031	Negative	0.25	Negative
